# Thymic Extranodal Marginal-Zone Lymphoma of Mucosa-Associated Lymphoid Tissue: Pathological Features, ^18^F-FDG PET/CT Findings and Prognosis in 12 Cases

**DOI:** 10.3389/fmed.2022.896647

**Published:** 2022-07-13

**Authors:** Shengbing Zang, Lei Liu, Junjie Bao, Min Xiong, Yumo Zhao, Suxia Lin, Xiaoping Lin

**Affiliations:** ^1^State Key Laboratory of Oncology in South China, Collaborative Innovation Center for Cancer Medicine, Sun Yat-sen University Cancer Center, Guangzhou, China; ^2^Department of Pathology, Sun Yat-sen University Cancer Center, Guangzhou, China; ^3^Department of Nuclear Medicine, Sun Yat-sen University Cancer Center, Guangzhou, China

**Keywords:** thymic neoplasm, MALT lymphoma, clinicopathologic features, positron emission tomography/computed tomography, ^18^F-FDG, prognosis

## Abstract

**Purpose:**

Primary thymic extranodal marginal zone lymphoma of mucosa-associated lymphoid tissue (MALT) lymphoma is a rare type of MALT lymphoma. We aim to investigate the clinicopathologic features, ^18^F-FDG PET/CT findings and outcomes for patients with primary thymic MALT lymphoma; to explore the correlation between metabolic parameters and immunohistochemical phenotypes.

**Methods:**

A retrospective single-center study enrolled 12 patients with primary thymic MALT lymphoma between 2010 and 2021. Nineteen ^18^F-FDG PET/CT scans were performed, and clinicopathologic and immunohistochemical characteristics, PET/CT imaging features, and outcomes were analyzed.

**Results:**

The male-to-female ratio was 1. The median age at diagnosis was 40 (range 31–68). The long diameter of the lesions ranged from 3.5 to 15.7. Histopathological examinations revealed that the normal thymic lobular architecture was effaced by a diffuse lymphoid infiltrate, but residual Hassall corpuscles could still be identified, mostly with CD20+, PAX5+, CD3-, CD23-, CD10-, BCL-6-, cyclin D1-, EBER- and low Ki-67. The gene rearrangement indicated that the IGH gene but not TCR gene was found in 7 patients. Six initial PET/CT scans showed a mean SUVmax of 6.8 (range, 3.1–12.4), a mean MTV = 40.0 (range, 6.7–81.4), and a mean TLG = 144.3 (range, 19.7–286.4). During the follow-up period, there was no death except for the patient with DLBCL who died 59 months after diagnosis of primary thymic MALT. No significant correlation between SUVmax and Ki-67 index was observed (r = 0.355, *P* > 0.05).

**Conclusion:**

Primary thymic MALT lymphoma should be considered in patients with multilocular cystic lesions with different degrees of ^18^F-FDG uptake in the anterior mediastinum. The results of this study showed no correlation between SUVmax and Ki-67 index.

## Introduction

Mucosa-associated lymphoid tissue (MALT) lymphoma is an indolent B-cell lymphoma, accounting for 5–8% of all non-Hodgkin lymphomas (NHLs) (1), which can present in any extranodal sites. It usually occurs in the stomach, salivary gland, lung, skin, thyroid, and breast (1–3). Different involvement sites may relate to different prognoses and clinical performance, leading to different treatment approaches. Compared with gastric MALT lymphoma, extragastric MALT has a higher tendency to infiltrate beyond the primary site and may relate to a poorer prognosis (4, 5). In addition, some studies have shown that MALT lymphomas are associated with autoimmune disorders and chronic inflammation, especially in extra-gastric origin (1, 6).

Primary thymic MALT lymphoma is an extremely rare type of MALT lymphoma. It was first reported by Isaacson et al. in 1983 as a new subtype of B-cell lymphoma (7), which usually has no obvious clinical symptoms. Most people find an anterior mediastinal mass incidentally on physical examination. Radiographically, thymic MALT lymphoma is often misdiagnosed as thymoma or other benign diseases. The diagnosis should be established by histopathology of the biopsied sample (8, 9). The role of ^18^F-fluorodeoxyglucose (FDG) positron emission tomography/computed tomography (PET/CT) of MALT lymphoma has been reported previously (10–13). However, most of them were confined to common sites of MALT, such as the stomach, salivary gland, and thyroid.

Recently, there have been a few reports on primary thymic MALT lymphoma, mainly involving clinical, pathological and immunohistochemical features (14, 15). However, to the best of our knowledge, few published studies have analyzed the PET/CT performance of primary thymic MALT lymphoma and its correlations with pathological features and prognosis.

Therefore, in this article, we aim to investigate the clinicopathologic features, ^18^F-FDG PET/CT findings, and outcomes for patients with primary thymic MALT lymphoma; to explore the correlation between ^18^F-FDG PET/CT metabolic parameters and immunohistochemical phenotypes.

## Patients

This study retrospectively analyzed 12 diagnosed primary thymic MALT patients at Sun Yat-Sen University Cancer Center from July 2010 to July 2021. The diagnosis of these cases was established histopathologically and immunohistochemically by two experienced hematopathologists (Szang and Slin), according to 2016 WHO criteria (9). Complete follow-up information for 12 patients was acquired until December 2021.

Eight patients underwent a total of 19 ^18^F-FDG PET/CT scans. Six of them underwent ^18^F-FDG PET/CT scan before treatment, and 4 patients had follow-up PET/CT scans. Two of them only had PET/CT scans after treatment. The remaining 4 cases were consultation cases that only had an initial computed tomography (CT) scan instead of PET/CT.

To evaluate the prognostic value of clinical data and PET parameters, overall survival (OS) was chosen as endpoints. OS was defined as the time from diagnosis of thymic MALT lymphoma to the date of death or last follow-up.

### Pathology and Gene Analysis

Formalin-fixed, paraffin-embedded (FFPE) tumor tissue sections were reviewed through hematoxylin-eosin (HE) staining, and regions containing more than 70% of tumor cells on unstained sections were selected for macrodissection by an expert pathologist (Lzhang). Immunohistochemistry was then performed in all tissue samples. The primary antibodies including antibodies against B-cell antigens (CD20 and PAX5), plasm cell antigens (CD38 and CD138), T-cell antigens (CD3, CD5, and CD43), germinal center markers (CD10 and Bcl6), CD23, cyclin D1, and the proliferation marker Ki-67 (Maixin Biotech, Fuzhou, China). EBV was detected in all cases by *in situ* hybridization according to the manufacturer's instructions (Maixin Biotech, Fuzhou, China).

Genomic DNA was extracted with all DNA samples quantified by NanoDrop 2000 (Thermo Scientific, Massachusetts, USA). The amplification of a short fragment of 110 pair base of human β-globin gene indicates the integrity of DNA for subsequent analysis. PCR amplifications were performed according to the manufacturer's instruction using a commercial BIOMED-2 multiplex PCR, IdentiClone Ig/TCR gene clonality assay (InVivo-Scribe Technologies, San Diego, CA, USA). After PCR amplification, the products were analyzed on an automated capillary electrophoresis system (ABI 3100, Applied Biosystems, Foster City, Calif) with GeneScan software (Applied Biosystems). Gene rearrangement was determined by 1–2 allele peaks in the region targeted by primers, with a peak height at least 2.5 times the background polyclonal peak. Clone peaks that did not meet the above criteria were considered negative.

### ^18^F-FDG PET/CT Protocol

All patients fasted for 5 to 6 h before ^18^F-FDG injection. No patient had diabetes and all had blood glucose less than 200 mg/dL (11.1 mmol/L). PET/CT scans were performed using an integrated PET/CT scanner (Discovery ST, GE Healthcare, Waukesha, Wis, USA; or Biograph mCT, Siemens Healthcare, Henkestr, Germany). Image data were acquired 60 ± 10 min after the ^18^F-FDG administration (3.7 ± 0.37 MBq/kg body weight). CT scans of the whole body including the skull to the mid-thigh were obtained in an arm-up position by Discovery ST (automatic tube current modulation, tube voltage 140 kV, rotation time 0.8 s, pitch 1.0, field of view 50 cm, collimation 16 × 1.25 mm, slice thickness 3.75 mm) were reconstructed in a 512 × 512 matrix; scans by Biograph mCT (tube current 80–200 mAs, voltage 120 kV, rotation time 0.5 s, pitch 1.0, field of view 50 cm, collimation 32 × 1.25 mm, slice thickness 3 mm) were reconstructed in a 512 × 512 matrix. The acquisition time per bed position of emission images was for 3 min in two-dimensional (2D) mode with Discovery ST, and 1.5–2 min in three-dimensional (3D) mode with Biograph mCT. The PET images were reconstructed with a slice thickness of 3.25 mm (2D) in a 128 × 128 matrix or with 2 mm (3D) in a 200 × 200 matrix, using the Ordered Subsets Expectation Maximization (OSEM) iterative image reconstruction method. PET, CT, and fused PET/CT images were generated for review on Xeleris computer workstation (GE Medical Systems).

### PET/CT Analyses

All PET/CT readings were completed by two experienced senior nuclear medicine physicians, and the conclusions were sometimes judged by the third senior nuclear medicine physician. A positive PET result was defined as a higher uptake of ^18^F-FDG than surrounding normal tissue, excluding physiological uptake (16). Outline the region of interest (ROI) of the patient's lesion and the maximum standardized uptake value (SUV_max_), tumor metabolic volume (MTV), and total lesion glycolysis (TLG) of the lesion were automatically measured on the workstation. MTV was automatically calculated as the lesion volume of the volumes-of-interest (VOI) using the 41% maximum standardized uptake value threshold. Then we manually subtracted the outlined normal tissue, if any. TLG was calculated as the summation of individual MTV multiplied by the SUVmean of every lesion.

### Statistical Analysis

Quantitative data conforming to normal distribution were expressed as mean ± standard deviation (SD). The statistical comparison of Ki-67 in different SUVmax groups was analyzed by Wilcoxon rank sum test. Linear regression was used to analyze the correlation of SUV_max_ with Ki-67 index in 6 patients with thymic MALT lymphoma who underwent initial PET/CT. We further analyzed the correlation between MTV, TLG and Ki-67 indices in four of these patients. All statistical analyses were performed using SPSS 25.0 (IBM, Armonk, USA). A *P* value of less than 0.05 is considered statistically significant.

## Results

### Clinical Characteristics and Follow-Up

Clinical characteristics of the 12 patients are summarized in Table 1. Half patients were male, which is not consistent with the previous reports that there is a female predominance (1, 2). The median age at diagnosis was 40 (range 31–68), which tend to be about 10–20 years younger than the patients in the previous studies (1, 2). Only 1 patient was older than 60 years. All patients (12/12) were presented with low stage (I or II) disease, where no bone marrow infiltration has been identified. Eight cases were asymptomatic and were incidentally diagnosed with CT scans, while three cases were found to have a mediastinal mass due to dyspnea accompanied by cough. One patient with a 4-year history of diffuse large B cell lymphoma in the tonsil was diagnosed with a CT scan. However, there was no direct evidence for a histogenetic link with the previous DLBCL. Three patients presented with either arthritis, systemic lupus erythematosus (SLE), or classic Hodgkin lymphoma (CHL). Five patients were treated surgically without chemotherapy or radiotherapy. Seven patients received chemotherapy or chemotherapy plus radiotherapy.

**Table 1:** Clinic and biologic characteristics of the 15 cases with thymic MALT lymphoma.

**Characteristics**	***N* = 12**
Gender (Male/Female)	6/6
Stage (I or II/III or IV)	12/0
Age (years)	40 (31-68)
Follow up (months)	25 (3-137)
Symptom (%)	
No symptom	8 (66.7%)
Dyspnea	3 (25.0%)
Arthralgia	1 (8.3%)
Complication (%)	
No complication	8 (66.7%)
Arthritis	1 (8.3%)
SLE	1 (8.3%)
CHL	1 (8.3%)
DLBCL	1 (8.3%)
Treatment	
Surgery	5 (41.7%)
Surgery + Chemotherapy	3 (25.0%)
Chemotherapy + Radiotherapy	4 (33.3%)
Survival	
Yes	12 (100%)
No	0 (0%)

*SLE, a systemic lupus erythematosus; CHL, classic Hodgkin lymphoma; DLBCL, diffuse large B cell lymphoma*.

With a median follow-up of 25 months (range: 3–137), there was no death except for the patient with DLBCL who died 59 months after diagnosis of primary thymic MALT.

### Radiography, Pathology, and Gene Rearrangement

All CT scan showed a hypodense mass in front of the ascending aorta and pulmonary artery, and all the lesions were clearly demarcated from the mediastinal vessels and heart, and there was no surrounding tissue involvement. The long diameter of the lesions ranges from 3.5 to 15.7 (mean ± SD, 6.7 ± 3.7) (Table 2). Crossly, most tumors were encapsulated and consisted of solid grayish-white fleshy tissue interspersed with multiple, variable-sized cysts. The cysts contained bloody or clear fluid and were surrounded by adipose tissue. Thymic MALT lymphoma and the multilocular thymic cyst are very similar in appearance and therefore can be easily misdiagnosed.

**Table 2:** Immunophenotype and gene alterations of the 15 cases with thymic MALT lymphoma.

**Case No**.	**CD20/PAX5**	**CD3/CD23/CD** **10/BCL-** **6/cyclin D1**	**CD5**	**CD43**	**CD38**	**CD138**	**Ki-67**	**EBERs**	**Gene**
**1**	**+/+**	-/-/-/-/-	-	-	-	-	8%	-	IGHR (+)
**2**	**+/+**	-/-/-/-/-	+	-	+	+	10%	-	IGHR (+)
**3**	**+/+**	-/-/-/-/-	-	N/A	-	-	10%	-	IGHR (+)
**4**	**+/+**	-/-/-/-/-	-	-	-	-	5%	-	N/A
**5**	**+/+**	-/-/-/-/-	-	+	-	-	8%	-	N/A
**6**	**+/+**	-/-/-/-/-	-	-	-	-	20%	-	IGHR (+)
**7**	**+/+**	-/-/-/-/-	-	+	+	+	8%	-	IGHR (+)
**8**	**+/+**	-/-/-/-/-	-	+	-	-	10%	-	N/A
**9**	**+/+**	-/-/-/-/-	-	-	-	-	10%	-	IGHR (+)
**10**	**+/+**	-/-/-/-/-	-	-	-	-	12%	-	N/A
**11**	**+/+**	-/-/-/-/-	-	+	-	-	3%	-	IGHR (+)
**12**	**+/+**	-/-/-/-/-	-	N/A	-	-	15%	-	N/A
**+/Total**	**12/12**	**0/12**	**1/12**	**4/10**	**2/12**	**2/12**	**12/12**	**0/12**	**7/7**

*IGHR, immunoglobulin heavy chain rearrangement; N/A, not available*.

**Figure 1:**
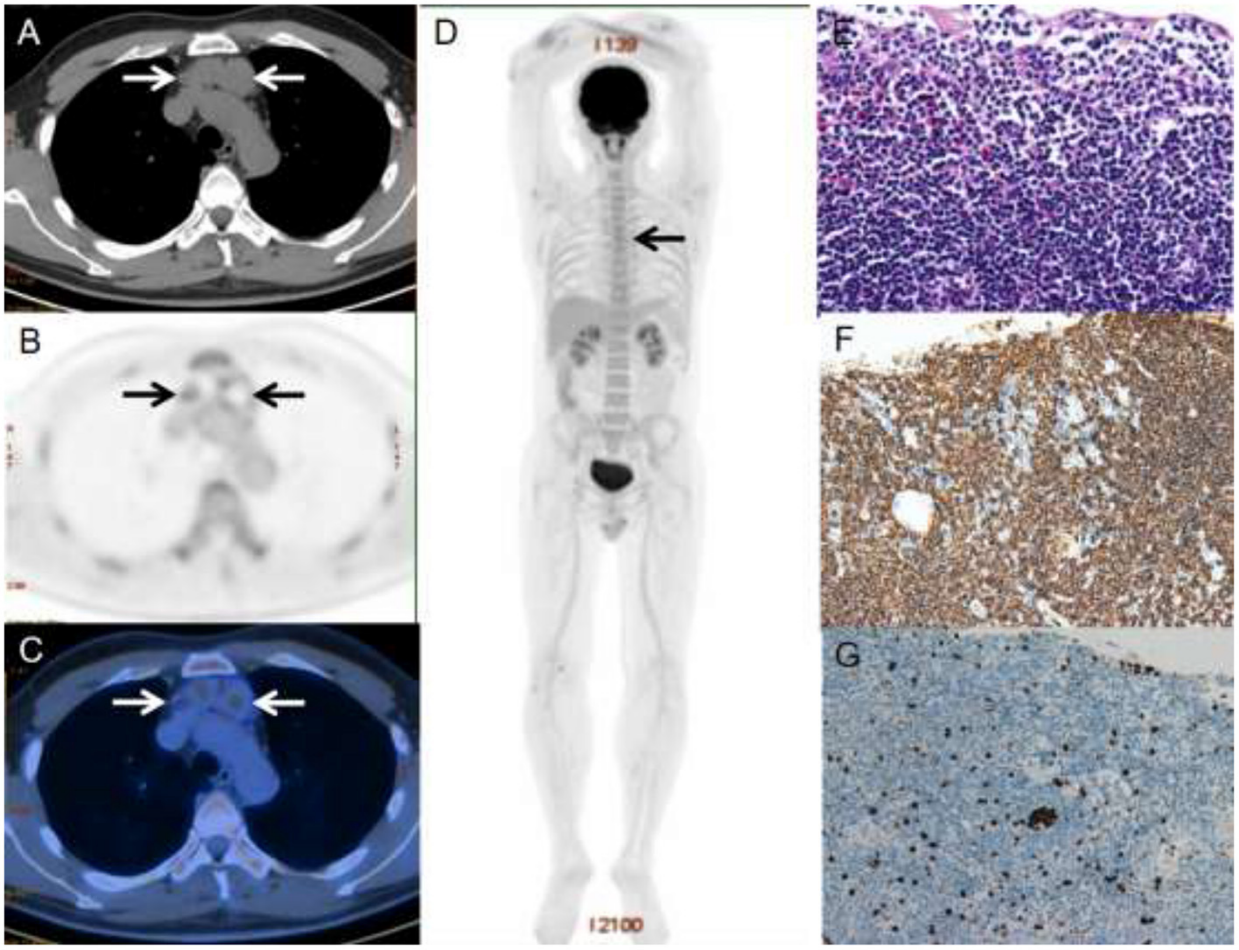
A 40-year-old male with primary thymic MALT lymphoma. PET/CT revealed multilocular cystic nodules with moderate ^18^F-FDG uptake (SUV_max_ = 5.0) in the anterior mediastinum, which was measured 2.6 × 3.6 cm in size **(A–C)**. Three-dimensional maximum intensity projection image **(D)** shows mild ^18^F-FDG-avid lesion in the anterior mediastinum (black arrows). HE staining revealed diffuse infiltrate of lymphoid cells replacing thymus tissue and all the cysts wall remained clear margin **(E)**. The immunophenotypic examination showed B-cell phenotype (CD20 positive) **(F)**. Ki-67 was 3%~5% **(G)**.

Under a microscope, the normal thymic lobular architecture was effaced by a diffuse lymphoid infiltrate, but residual Hassall corpuscles can still be identified (Figure 1E). The cyst wall was covered by squamous epithelium, which was scattered with medium-sized lymphocytes consisting of monocyte-like B cells and centrocyte-like cells, with occasional plasma cells.

The immunophenotype results are summarized in Table 2. All cases were positive for B-cell markers (CD20 and PAX5) (Figures 1F,G) and negative for CD3, CD23, CD10, BCL-6 and cyclin D1. Four of 10 patients were positive for CD43, and 1 of 12 patients were positive for CD5. All 12 patients with available results were negative for EBER. The tumor cells in two cases expressed CD38 and CD138, indicating plasma cell differentiation. In addition, the proliferation marker Ki-67 was low in all cases, with around 75% of patients (9/12) showing Ki-67 were no more than 10%.

We also performed IGH and TCR gene rearrangement tests on seven tissues, and clonal immunoglobulin genes were detected in all seven patients, but no clonal TCR gene rearrangements were found in any of them.

### PET/CT Findings

At six initial PET/CT scans, all patients presented with multiple cystic nodules in the anterior mediastinum with varying degrees of ^18^F-FDG uptake and the SUV_max_ of these lesions ranges from 3.1 to 12.3 (mean ± SD, 6.8 ± 3.4) (Figures 1A–D). Volumetric metabolic parameters were obtained for only 4 patients. The MTV of these lesions ranges from 6.7 to 81.4 (mean ± SD, 40.0 ± 31.7). The TLG of these lesions range from 19.7 to 286.4 (mean ± SD, 144.3 ± 112.2). Three patients had mild and moderate uptake of ^18^F-FDG, which was lower than or close to liver uptake, and the remaining patients had high uptake of ^18^F-FDG, which was significantly higher than liver uptake (Table 3).

**Table 3:** CT or PET/CT features and staging in patients with thymic MALT lymphoma.

**No**.	**Sex**	**Age (years)**	**Imaging** **modality**	**SUV_**max**_**	**MTV**	**TLG**	**Length to diameter (cm)**	**Border**	**With cyst**	**Peripheral organs invasion**	**Stage**
1	M	40	PET/CT	5.0	6.7	19.7	3.6	WD	+	-	I
2	M	31	PET/CT	8.9	81.4	286.4	6.5	WD	+		II
3	M	36	CT	N/A	NA	N/A	3.5	WD	+	-	I
4	F	31	PET/CT	3.1	NA	N/A	15.7	WD	+	-	II
5	M	47	PET/CT	6.6	45.0	166.7	4.8	WD	-	-	II
6	F	38	CT	N/A	NA	N/A	5.0	WD	+	-	II
7	F	52	PET/CT	12.3	27.2	104.6	5.1	ID	+	-	II
8	F	34	CT	N/A	NA	N/A	N/A	N/A	N/A	N/A	II
9	F	40	PET/CT	4.6	NA	N/A	8.0	N/A	N/A	N/A	II
10	M	49	CT	N/A	NA	N/A	N/A	N/A	N/A	N/A	I
11	F	32	CT	N/A	NA	N/A	8.1	N/A	N/A	N/A	II
12	M	68	CT	N/A	N/A	N/A	N/A	N/A	N/A	N/A	II

*SUV_max_, the maximum standardized uptake value; MTV, tumor metabolic volume; TLG, Total lesion glycolysis; N/A, not available; Border, WD, well-defined*.

All patients' lesions were staged according to the Ann Arbor staging system for lymphoma; Table 3 shows the staging of the 12 patients. Two patients had extra-thymic lymph node infiltration, and the remaining patients had lesions confined to the anterior mediastinum. Involvement of extra-thymic non-lymph node was not found in our cases.

**Figure 2:**
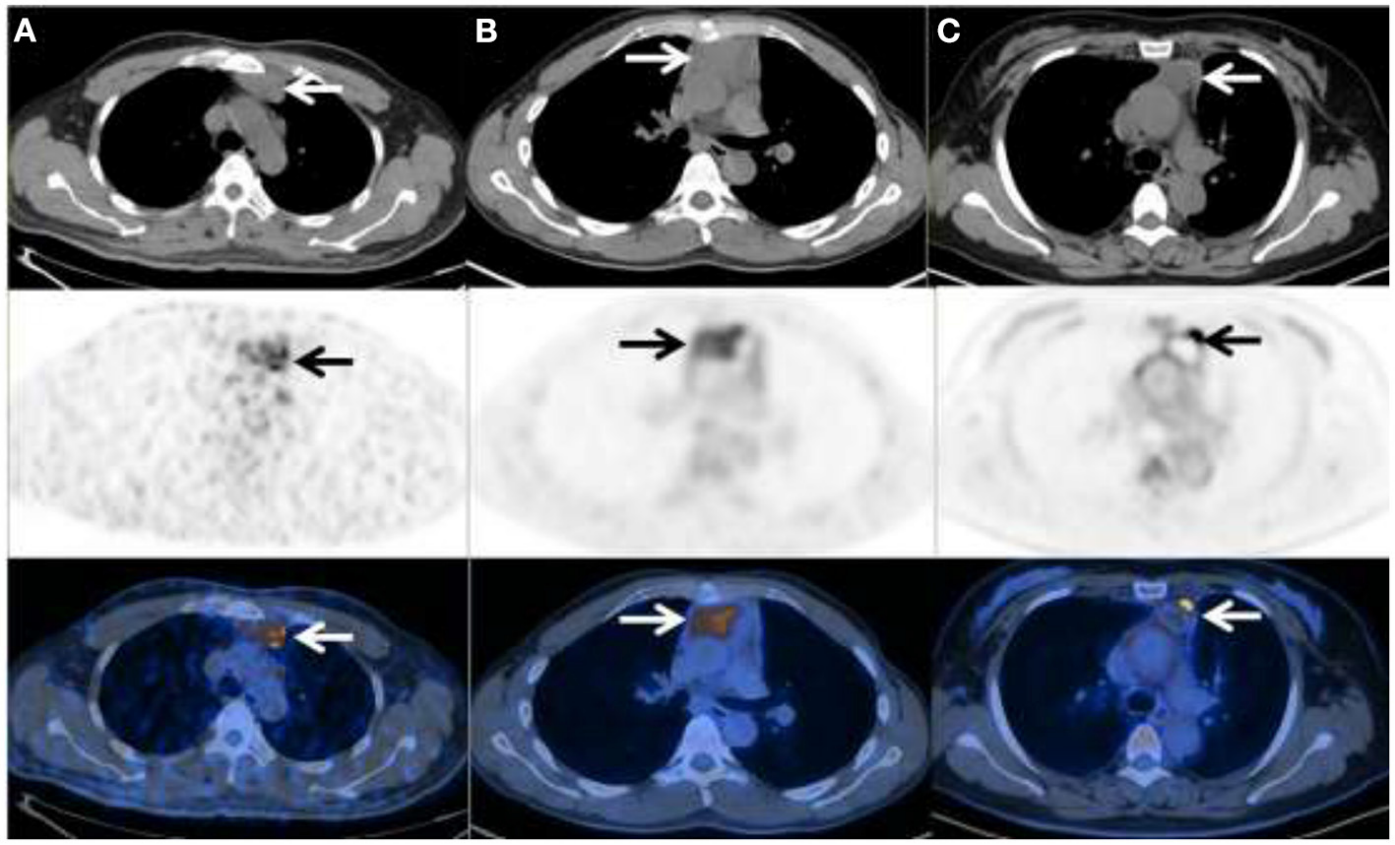
^18^F-FDG PET/CT in three patients with primary thymic MALT lymphoma. **(A)** Male, 47y, Ki-67 = 8%, SUV_max_ = 7.17, TLG = 99.85, MTV = 24.14 **(B)** Male, 31y, Ki-67 = 10%, SUV_max_ =6.08, TLG = 280.93, MTV = 80.02 **(C)** Female, 47y, Ki-67 = 8%, SUV_max_ = 12.34, TLG = 35.54, MTV = 5.14.

After treatment, follow-up PET/CT scans were performed in 3 patients, 2 of which achieved complete remission (Figure 2) while the other patient progressed due to concurrent DLBCL.

### The Correlation Between Metabolism Index (SUV_max_, TLG and MTV) and Ki-67 Index

**Figure 3:**
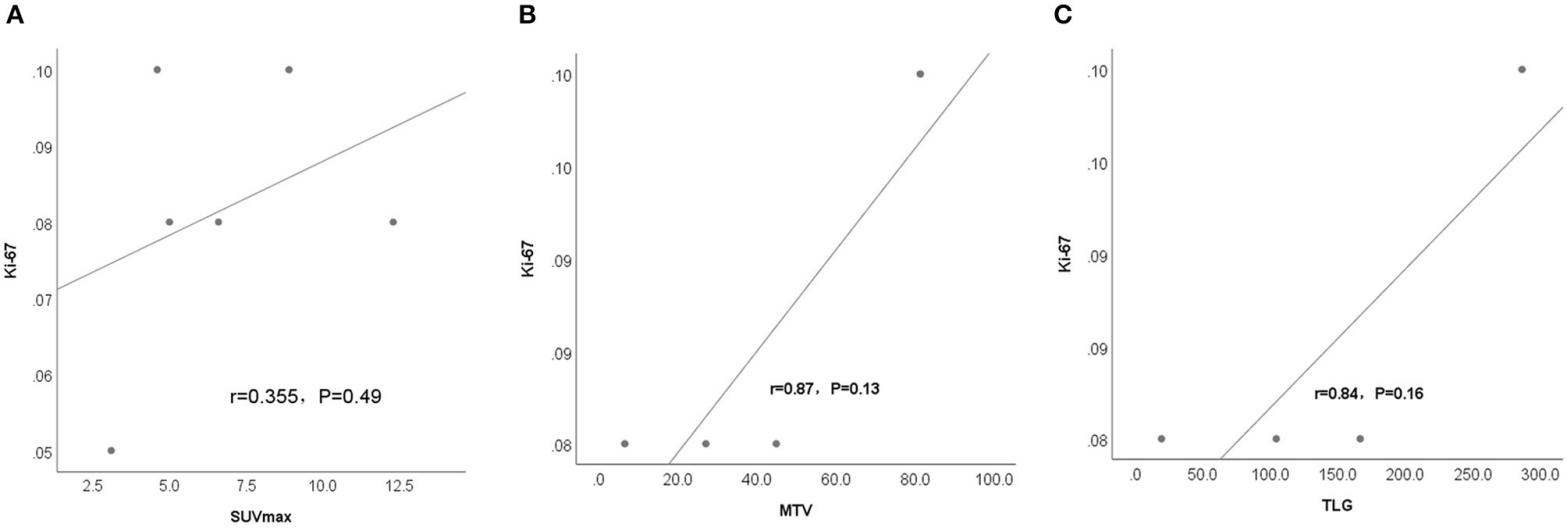
Linear regression analysis shows no significant correlation between metabolism parametrix, including **(A)** SUV_max_, **(B)** MTV, and **(C)** TLG in the thymic lesions of the 6 patients with thymic MALT lymphoma and proliferation index (Ki-67%).

The correlation between the SUV_max_, TLG and MTV of thymic lesions obtained on ^18^F-FDG PET/CT scans and the expression of Ki-67 on immunohistochemistry was tested by linear regression analysis, which shows no significant correlation between them in patients with thymic MALT lymphoma (r = 0.355, *P* = *P* = 0.49), (r = 0.87, *P* = 0.13), (r = 0.84, *P* = *P* = 0.16) (Figure 3). The whole group was divided into two SUVmax subgroups, based on the median SUVmax of the six patients. Ki-67 between different subgroups was compared using the Wilcoxon rank sum test, which showed no statistically significant differences between groups (Z=-0.471, *P* = 0.70), but the higher SUVmax showed a trend toward higher KI-67 (Mean rank: high SUVmax vs. low SUVmax, 3.83 vs. 3.17).

## Discussion

In this study, we investigated the clinicopathologic features, ^18^F-FDG PET/CT findings, and outcomes for patients with primary thymic MALT lymphoma. The lesions are usually confined to thymic tissue, present with multiple cystic nodules, sometimes with infiltration of extrathymic lymph nodes. The immunophenotype showed positive B-cell markers (CD20 and PAX5) and negative for EBER in all patients. The gene rearrangement indicated that IGH gene but not TCR gene was found in 7 patients. Our results indicated that thymic MALT lymphoma was a disease of ^18^F-FDG avid, and ^18^F-FDG PET/CT proved helpful for the diagnosis, staging and therapeutic efficacy evaluation of thymic MALT lymphoma. In addition, there has been no significant correlation between SUV_max_ of thymic lesions and Ki-67 in the patients.

Since the first report of primary thymic MALT lymphoma in 1983, 113 cases have been reported worldwide, and this number has increased to 125 with our study. Among them, 30 were male, and 95 were female, mostly Asian. Thymic MALT lymphoma is associated with autoimmune diseases, especially Sjögren syndrome (17), which was inconsistent with our study; only one (1/12) patient developed Sjögren's syndrome. Compliance with previous studies (18), the clinical prognosis of patients with thymic MALT lymphoma in our study was favorable, except for one (1/12) patient with DLBCL who progressed, which implied DLBCL is a poor prognostic factor for MALT lymphoma (19).

The final diagnosis of thymic MALT lymphoma still depends on pathology and immunohistochemistry. Consistent with previous reports (17, 20), the typical pathology features are cystic or cystic-solid tissue. In this study, tumor cells from thymic MALT lymphoma expressed B-cell markers (CD20 and PAX5), which demonstrates that MALT lymphomas originate in the marginal zone (MZ) of B cells. Some studies mentioned that these MZ B cells seem to be capable of differentiating into plasma cells (21). In addition, these MZ B cells have rearranged and mutated immunoglobulin heavy and light chain genes (10). In our study, clonal immunoglobulin genes were identified in all 7 cases. Our study is the first to explore the ^18^F-FDG PET/CT features of thymic MALT lymphoma.

^18^F-FDG PET/CT has been proven to be a reliable tool for initial staging, response evaluation, and follow-up surveillance in Hodgkin disease and multiple non-Hodgkin lymphomas (22–24). However, studies on ^18^F-FDG PET/CT in MALT lymphoma are insufficient with inconsistent results. Pretreatment ^18^F-FDG PET/CT showed variation in ^18^F-FDG avidity according to site involvement (2), while some studies reported negative ^18^F-FDG uptake at MALT lymphomas' involved sites (2, 25, 26). Only few prior studies have mentioned primary thymic MALT lymphoma due to the rarity. Hence, the role of PET/CT in MALT lymphoma requires further investigation.

We found that the uptake of ^18^F-FDG was significantly higher in most of the lesions (4/6) than that of the liver, and PET/CT detected lymph node infiltration in two patients. Multilocular cystic appearance is not common in lymphoma (27). Thus, it is easy to be misdiagnosed as thymoma or other benign diseases. Evaluating treatment response of MALT lymphoma by ^18^F-FDG PET/CT had been demonstrated to be useful in previous studies (3). Our study mainly showed that PET/CT may be helpful in monitoring treatment response for patients with thymic MALT lymphoma. Overall, our results suggest that ^18^F-FDG PET/CT is useful in providing the tumor burden, staging, detecting lymph node involvement, and evaluating treatment response of thymic MALT lymphoma.

Importantly, we found that Ki-67 of most patients with thymic MALT lymphoma in our cases were no more than 10%, which proved that the disease was indolent. Previously, the significant correlation of Ki-67 and SUV_max_ on PET/CT had been evaluated in extragastric MALT lymphoma but not thymus reported by Domenico Albano et al. (10). However, no significance of such was seen in the 6 cases in our study, which might be caused by the limited sample size.

The limitation of this study includes the retrospective and single-center design, limited sample size due to the rarity of the disease, and only a subset of patients had initial PET/CT, which was not conducive to our statistical analysis. Additionally, metabolic parameters such as SUV_max_, MTV and TLG were not fully covered in the study. Further studies with a large sample size and multiple centers and prospective studies are needed.

In summary, although rare, primary thymic MALT lymphoma should be considered in patients with multilocular cystic lesions with different degrees of FDG uptake in the anterior mediastinum on ^18^F-FDG PET/CT. ^18^F-FDG PET/CT is a useful tool for initial staging and evaluating treatment response of thymic MALT lymphoma. No correlation between SUV_max_ and Ki-67 index was observed in our study.

## Data Availability Statement

The original contributions presented in the study are included in the article/supplementary material, further inquiries can be directed to the corresponding authors.

## Ethics Statement

The studies involving human participants were reviewed and approved by Sun Yat-sen University Cancer Center. Written informed consent for participation was not required for this study in accordance with the national legislation and the institutional requirements.

## Author Contributions

SZ and XL: conceptualization, methodology, resources, and supervision. JB and MX: formal analysis and investigation. JB, MX, and LL: writing—original draft preparation. LL and YZ: writing—review and editing. SL and XL: technical support. All authors read and approved the final manuscript.

## Conflict of Interest

The authors declare that the research was conducted in the absence of any commercial or financial relationships that could be construed as a potential conflict of interest.

## Publisher's Note

All claims expressed in this article are solely those of the authors and do not necessarily represent those of their affiliated organizations, or those of the publisher, the editors and the reviewers. Any product that may be evaluated in this article, or claim that may be made by its manufacturer, is not guaranteed or endorsed by the publisher.
